# The clinical features and image characteristics of Meniere's disease patients with endolymphatic hydrops confirmed by enhanced magnetic resonance imaging

**DOI:** 10.1016/j.bjorl.2021.07.009

**Published:** 2021-10-17

**Authors:** Wei Chen, Xiao Wu, Yue Geng, Naier Lin, Yan Sha

**Affiliations:** a Eye & ENT Hospital of Fudan University, Department of Radiology, Shanghai, China; b Fudan University, Shanghai Institute of Medical Imaging, Shanghai, China; c Eye & ENT Hospital of Fudan University, Department of Otolaryngology, Shanghai, China

**Keywords:** Meniere’s disease, Endolymphatic hydrops, Clinical feature, Image characteristic, Magnetic resonance imaging

## Abstract

**Objectives:**

To analyze and summarize the clinical features and image characteristics of Meniere's Disease (MD) patients with Endolymphatic Hydrops (EH) confirmed by enhanced Magnetic Resonance Imaging (MRI).

**Methods:**

252 MD patients with EH confirmed by MRI were enrolled. All patients met the diagnostic criteria for MD and underwent intravenous gadolinium injection. After 4 h, MR examinations were performed. The Nakashima grading standard was used to classify EH and evaluate its correlation with clinical features.

**Results:**

Different degrees of EH were shown in all MD patients, and 157 of the 252 (62.3%) patients showed significant EH, 95 of the 252 (37.7%) patients showed mild EH. Only 89 (35.3%) met the diagnostic criteria for definite MD, and the remaining 163 (64.7%) patients met the diagnostic criteria for probable MD. Compared with patients with unilateral EH, the symptoms of the first affected ear of patients with bilateral EH were more serious. The degree of EH was related to the degree of hearing loss (*p* < 0.05).

**Conclusion:**

MRI with intravenous gadolinium injection can provide a better assessment of EH in MD patients. The clinical features of MD patients with EH confirmed by enhanced MRI did not fully meet the existing diagnostic criteria for definite MD. Including the diagnosis of EH in the diagnostic criteria of MD can increase the diagnosis rate of MD. The degree and distribution of EH may be related to the degree of hearing loss.

**Level of evidence:**

4.

## Highlights

• 3D-real IR MRI can provide a better assessment of EH in MD patients.
• Including EH in the diagnostic criteria can increase the MD diagnosis rate.
• The EH degree or distribution may be related to the degree of hearing loss.

## Introduction

Meniere’s Disease (MD) is an idiopathic inner ear disease whose cause is still unclear, named after the French physician Prosper Meniere first proposed in 1861. Hallpike and Cairns^1^ reported that the major pathological change of the disease was Endolymphatic Hydrops (EH) of the membranous labyrinth in 1938. This finding had been confirmed by many scholars. The clinical symptoms of MD are recurrent rotating vertigo with fluctuating hearing loss, tinnitus, and ear fullness. At present, the diagnosis of MD mainly depends on a range of clinical symptoms but lacks objective imaging evidence. In 2007, Nakashima et al.^2^ applied transtympanic gadolinium contrast MRI technology to see EH in the cochlea or (and) vestibule of MD patients. This was the first time that the inner ear gadolinium imaging technology had been applied to the MD patients to observe EH.

The clinical manifestations of MD are often atypical, and vestibular migraine, psychogenic vertigo, benign paroxysmal positional vertigo, etc. can also be manifested as vertigo symptoms similar to MD,^3^ which indirectly increases the difficulty of the diagnosis of MD. Although EH is the recognized pathological basis of MD, in addition to MD, traumatic diseases of the inner ear, sudden deafness, low-frequency sensorineural hearing loss, recurrent peripheral vestibular disease,^4^ semicircular canal dysplasia,^5^ semicircular canal fissure syndrome, and vestibular aqueduct syndrome^6^ can also be manifested as EH. Therefore, the diagnosis of MD needs to combine clinical manifestations and imaging characteristics at the same time.

In this study, we further analyzed and summarized the clinical features and image characteristics of MD patients with EH confirmed by MRI in order to provide some clinical and imaging evidence for the accurate diagnosis of MD.

## Methods

### Patients

From July 2018 to September 2020, 252 MD patients (208 unilateral cases and 44 bilateral cases, totaling 296 affected ears) who underwent MR scans via intravenous gadolinium injection of the inner ear at our hospital were enrolled in this study, including 113 men and 139 women, with an average age of 48.75 years (11–79 years). The clinical features such as age, gender, affected side, disease course, hearing loss and other symptoms such as vertigo, tinnitus and ear fullness were investigated. All patients met the diagnostic criteria for definite or probable MD and had varying degrees of EH in the inner ear. The diagnostic standard was based on the 2015 Equilibrium Committee Amendment^7^ for the diagnosis of MD (Table 1). The clinical data of all patients were complete, the external auditory canal and tympanic membrane were intact confirmed by otoscope examination, other ear organic diseases were excluded, there was no history of contrast agent allergy, and non-pregnant women. All patients had clinical manifestations of vertigo and ear symptoms, and EH was confirmed by inner ear MRI. Patients with typical migraine, sudden deafness, central or benign paroxysmal positional vertigo were excluded. All patients voluntarily accepted the enhanced MRI examination and signed the informed consent form. The medical ethics committee of our hospital had approved this research (2020056).

**Table 1:** The amended 2015 criteria for diagnosis of Meniere’s disease.^7^

Definite	Two or more spontaneous episodes of vertigo, each lasting 20 min to 12 h
	Audiometrically documented low- to midfrequency sensorineural hearing loss in 1 ear, defining the affected ear on at least 1 occasion before, during, or after 1 of the episodes of vertigo
	Fluctuating aural symptoms (hearing, tinnitus, or fullness) in the affected ear
	Not better accounted for by another vestibular diagnosis

Probable	Two or more episodes of vertigo or dizziness, each lasting 20 min to 24 h
	Fluctuating aural symptoms (hearing, tinnitus, or fullness) in the affected ear
	Not better accounted for by another vestibular diagnosis

### Pure-tone audiometry

Pure-Tone Audiometry (PTA) thresholds at all conventional frequencies (125–8000 Hz) were tested by applying a GSI 61 audiometer (Grason-Stadler, Eden Prairie, MN). Furthermore, the average hearing threshold of all frequencies was calculated.

### Image acquisition

In our study, all MD patients underwent intravenous injection with the double dose (0.4 mL/kg) of gadolinium (ProHance, Bracco Imaging Italia SRL, Milano, Italy). After 4 h, MR examinations were performed on all patients. All examinations were applied by a 3 T MRI scanner (Magnetom Verio, Siemens Healthineers, Erlangen, Germany) with a 8-channel ear-surface phased-array coil. The T2 Sampling Perfection with Application-optimized Contrasts by using different flip angle Evolutions (SPACE) and three-dimensional Inversion-Recovery sequence with real reconstruction (3D-real IR) sequences were applied in collecting images. The parameters of T2 SPACE and 3D-real IR sequences were shown in Table 2.

**Table 2:** The parameters of T2 SPACE and 3D-real IR sequences.

Parameter	SPACE	3D-real IR
Slice thickness	0.6 mm	0.6 mm
Repetition time	1000 ms	6000 ms
Echo time	132 ms	181 ms
Inversion time	/	1850 ms
Flip angle	120°	180°
Matrix size	384 × 384	768 × 768
Field of view	200 mm × 200 mm	160 mm × 160 mm
Scan time	2 min 44 s	15 min 20 s

### Endolymphatic hydrops evaluation

The gadolinium flows into the perilymphatic space of the inner ear through the blood-labyrinth circulatory system, which makes the perilymphatic space appear as obvious high signal on the MRI. Meanwhile, due to the existence of the barrier between endolymph and perilymph, the gadolinium contrast agent cannot enter the endolymphatic space, so the endolymphatic space shows obviously low signal on the MRI. This imaging principle can be used to distinguish endolymph from perilymph, and then to determine whether there is EH. In this research, we applied the method reported by Nakashima et al.^8^ for the EH grading (Table 3). Based on the double-blind principle and the grading method displayed on Table 3, two radiologists with eleven and thirteen years of working experience respectively in the diagnosis of inner ear diseases independently browsed and evaluated the images. If there were differences of result, the final outcome depended on the consensus reached by both sides.

**Table 3:** The evaluation criteria for Endolymphatic Hydrops (EH) after intravenous gadolinium injection.^8^

EH Grade	Vestibule (area ratio^a^)	Cochlea	EH evaluation
None	≤33.3%	No displacement of Reissner's membrane	Negative
Mild	>33.3%, ≤50%	Displacement of Reissner's membrane. Area of cochlear duct ≤ area of the scala vestibuli	Positive
Significant	>50%	Area of the cochlear duct exceeds the area of the scala vestibuli	Positive

a Area ratio = EH area/(EH area + perilymphatic area).

### Statistical analyses

The IBM SPSS (Version 22; IBM, Armonk, New York) was used to handle all data. The independent sample *t*-test and the χ2-test were used for comparison between groups. And *p* < 0.05 was considered statistically significant.

## Results

### MRI results

**Figure 1:**
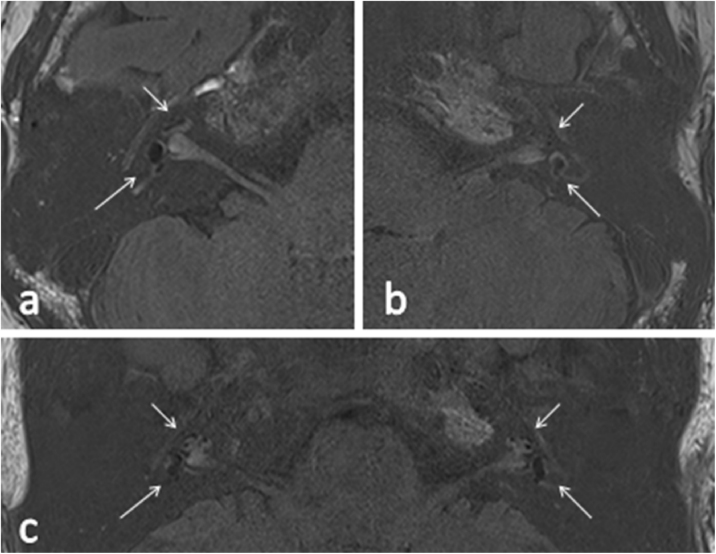
(a) A 53-year-old female with right Meniere’s disease, 3D-real IR MRI was performed after intravenous gadolinium injection in double doses. The long arrow indicated significant vestibular endolymphatic hydrops existed, and the short arrow indicated significant cochlear endolymphatic hydrops existed. (b) A 33-year-old male with left Meniere’s disease, 3D-real IR MRI was performed after intravenous gadolinium injection in double doses. The long arrow indicated mild vestibular endolymphatic hydrops existed, and the short arrow indicated mild cochlear endolymphatic hydrops existed. (c) A 29-year-old male with bilateral Meniere’s disease, 3D-real IR MRI was performed after intravenous gadolinium injection in double doses. The long arrow indicated significant vestibular endolymphatic hydrops existed, and the short arrow indicated significant cochlear endolymphatic hydrops existed.

The 252 patients enrolled in our group all obtained good images of the inner ears via MR scans with intravenous gadolinium injection. Different degrees of EH were shown in the vestibule or different turns of the cochlea in the affected ears of all MD patients, and Fig. 1 showed the conditions of EH in different patients. 157 of the 252 (62.3%) patients showed significant EH, 95 of the 252 (37.7%) patients showed mild EH. 208 patients (82.5%) showed EH in unilateral ear, and 44 patients (17.5%) showed EH in bilateral ears.

**Figure 2:**
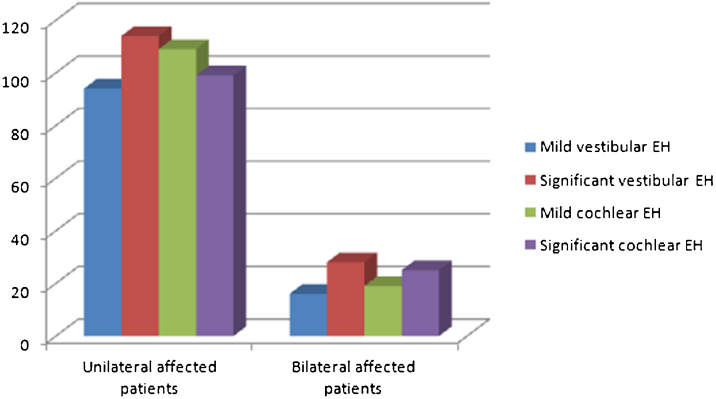
The specific distribution of affected ears with different degrees of Endolymphatic Hydrops (EH) in unilateral and bilateral patients.

Among the 208 patients with unilateral EH, 94 cases (45.2%) had mild vestibular EH, 114 cases (54.8%) had significant vestibular EH; 109 cases (52.4%) had mild cochlear EH, and 99 cases (47.6%) had significant cochlear EH. The specific distribution of affected ears with different degrees of EH in unilateral and bilateral patients were shown in Fig. 2. Among the first symptomatic ear in 44 bilateral EH patients, 16 cases (36.4%) had mild vestibular EH, 28 cases (63.6%) had significant vestibular EH; 19 cases (43.2%) had mild cochlear EH, and 25 cases (56.8%) had significant cochlear EH. The proportion of significant EH in the first symptomatic ear of 44 bilateral patients was higher than that in patients with unilateral EH (Vestibule: 63.6% vs. 54.8%; Cochlea: 56.8% vs. 47.6%), but the differences were not statistically significant (all *p* > 0.05) (Table 4).

**Table 4:** Comparison of the Endolymphatic Hydrops (EH) degree between unilateral EH patients and bilateral EH patients.

EH degree	Vestibule		Cochlea	
	Unilateral EH patients (n = 208)	Bilateral EH patients (n = 44)	Unilateral EH patients (n = 208)	Bilateral EH patients (n = 44)
Mild EH	94 (45.2%)	16 (36.4%)	109 (52.4%)	19 (43.2%)
Significant EH	114 (54.8%)	28 (63.6%)	99 (47.6%)	25 (56.8%)
χ^2^	3.192		3.542	
p	0.113		0.179	

### Clinical features

In the affected ears of 252 patients, the presence of EH was confirmed by enhanced MR scan. Among all patients, 113 were males and 139 were females, with an average age of 48.75 years (11–79 years). All patients showed dizziness and other ear symptoms. 115 cases (45.6%) had symptoms in the left ear, 93 cases (36.9%) had symptoms in the right ear, and 44 cases (17.5%) had symptoms in both ears. The specific clinical features were classified as follows:

1) Causes and accompanying symptoms: Among the 252 patients, the onset of vertigo had no obvious connection with the body position, and vertigo could occur when lying down, standing, sitting, or walking; 54 cases (21.4%) felt that the onset of vertigo was sometimes related to fatigue, bad mood, and weather changes; 198 cases (78.6%) felt that there was no obvious cause. Besides, 226 cases (89.7%) had nausea and vomiting during the onset of vertigo. 60 cases (23.8%) were accompanied by sensitivity to light, cold, and sound during the onset of vertigo, and 66 cases (26.2%) had some symptoms such as diarrhea, swelling of the head, numbness of the hands and feets, difficulty speaking, sound sensation and shoulder pain during the onset of vertigo, and all cases were not accompanied by typical migraine symptoms.
2) The ear symptoms: 252 patients all had low-medium frequency hearing loss. Among them, 29 cases reported that their hearing conditions were not significantly changed during the onset of vertigo compared with the intermittent period. Moreover, 243 cases (96.4%) had symptoms of tinnitus and/or ear fullness, of which 214 cases (84.9%) had more serious symptoms of tinnitus and/or ear fullness before and after the onset of vertigo, and the remaining patients felt that the symptoms did not change significantly during the onset of vertigo compared with the intermittent period.
3) Duration of vertigo: The duration of vertigo in each patient was variable. The duration of vertigo in 89 cases (35.3%) lasted 20 min – 12 h, and the duration of vertigo in 163 cases (64.7%) lasted 20 min – 24 h.
4) Frequency of vertigo attacks: In the early stage, dizziness usually occurred once every few years in some patients, and then it got gradually worse and could be manifested as once every few days to several years. When the onset was the most frequent, 25 cases (9.9%) occurred once every few days; 49 cases (19.4%) occurred once every few weeks; 60 cases (23.8%) occurred once every few months; 21 cases (8.4%) occurred once every few years; and 97 cases (38.5%) occurred once with uncertain frequency.
5) Genetic predisposition: Relatives (father or mother) of 22 patients (8.7%) had a positive history of vertigo. Besides, in 8 patients, their mothers, and grandmothers both had a positive history of vertigo. The remaining patients had no obvious family history and genetic predisposition.

### Correlation between EH and clinical features

The age, age of first onset, and course of disease of 208 patients with unilateral EH and 44 patients with bilateral EH were statistically analyzed. The results showed that the differences of the ages ([47.6 ± 12.5] years vs. [49.9 ± 15.1] years, *p* = 0.426), age of first onset ([41.3 ± 15.8] years vs. [43.3 ± 12.9] years, *p* = 0.271) and course of disease ([34.4 ± 13.1] moths vs. [43.6 ± 17.2] months, *p* = 0.173) between the unilateral patients and the bilateral patients were not statistically significant (Table 5).

**Table 5:** Comparison of the clinical features between unilateral Endolymphatic Hydrops (EH) patients and bilateral EH patients.

Clinical feature	Unilateral EH patients (n = 208)	Bilateral EH patients (n = 44)	t	*p*
Age (year)	47.6 ± 12.5	49.9 ± 15.1	−0.613	0.426
Age of first onset (year)	41.3 ± 15.8	43.3 ± 12.9	−1.382	0.271
Disease course (month)	34.4 ± 13.1	43.6 ± 17.2	−1.395	0.173

The average hearing thresholds of the MD patients with different levels of EH in the vestibule and cochlea had statistically significant differences (all *p* < 0.001), and the EH levels of the vestibule and cochlea were correlated with the degree of hearing loss (Table 6).

**Table 6:** Comparison of the Average Hearing Threshold (AHT) of vestibule and cochlea in Meniere's disease patients with different levels of Endolymphatic Hydrops (EH).

EH location	AHT (mild EH)	AHT (significant EH)	*p*
Vestibule	48.6 ± 13.3 (dB)	60.2 ± 11.7 (dB)	<0.001
Cochlea	50.5 ± 16.9 (dB)	63.8 ± 14.1 (dB)	<0.001

## Discussion

According to the latest statistical studies such as Bruderer et al.,^9^ the incidence of MD in recent years was about 13.1 per 100,000. Although more and more studies on MD were published in recent years, there is still no clear conclusion about its etiology. With the wide application of gadolinium contrast technology, EH was increasingly found in the inner ear of MD patients.^2^, ^10^ Meanwhile, in the animal experiments, membrane labyrinth swelling characterized by EH was considered to be the key pathological change of MD.^11^ Therefore, the presence or absence of EH had gradually become an important indicator for assisting the diagnosis of MD. However, in addition to MD, many other inner ear diseases can also be manifested as EH. Besides, due to the diverse clinical manifestations of MD, it is easy to be confused with other vertigo diseases such as migraine. Therefore, this study selected 252 patients whose clinical symptoms at least met probable MD and confirmed the presence of EH by inner ear MRI, and further analyzed their clinical features and imaging characteristics.

Our study showed that the occurrence of MD was not significantly related to gender. For the reports that the proportion of women in the diagnosis of MD was significantly higher than that of men, we speculated that this may be due to the misdiagnosis of migraine patients. The majority of MD groups were middle-aged and elderly, indicating that we should be cautious about the diagnosis of MD in minors and young people. Among the 252 MD patients, the onset of vertigo was mostly unrelated to changes in body position, and most of them had no obvious cause. Some patients experienced the onset of vertigo during sleep. Besides, 226 patients (89.7%) had nausea and vomiting during the onset of vertigo. Maybe we can consider antiemetic drugs for preventive treatment before the onset of the disease. 60 patients had the apprehensive symptoms such as fear of light, fear of cold, and fear of sound during the onset of the disease. Vestibular migraine was easily confused with MD.^12^ About 30% of patients with vestibular migraine had no headache symptoms when the onset of the disease, and the dizziness could last for several seconds or even days.^13^ Some studies believed that MD and vestibular migraine can exist at the same time, and vestibular migraine may be a factor that exacerbates the development of MD. But in patients with vestibular migraine, EH generally did not exist.^14^ Therefore, inner ear MRI can effectively distinguish the two.

Vertigo-related fluctuating low-medium frequency hearing loss, tinnitus, and ear fullness are characteristic manifestations of MD. The hearing examination showed that 252 patients all had low-medium frequency hearing loss. Moreover, 243 patients (96.4%) had symptoms of tinnitus and/or ear fullness. It can be seen that ear symptoms are an important factor in the diagnosis of MD, but the manifestations of tinnitus and ear fullness are diverse, so the diagnosis of MD cannot be completely dependent on the patient's self-reported ear symptoms. Meanwhile, our research showed that the duration and frequency of vertigo in different patients were not the same. Besides, our study found that in some MD patients, the onset of vertigo had a certain hereditary nature. This was consistent with the report of Requena et al.^15^ Relatives (father or mother) of 22 patients (8.7%) had a positive history of vertigo. In 8 patients, their mothers, and grandmothers both had a positive history of vertigo. This suggested that MD may have a certain degree of maternal inheritance. Regarding the hereditary characteristics of MD, further research is needed in the future.

The MRI results showed that compared with patients with unilateral EH, the symptoms of the first affected ear of patients with bilateral EH were more serious. There were no significant differences in the age, age of first onset, and course of disease between unilateral EH patients and bilateral EH patients. In addition, we found that the degree of EH was significantly positively correlated with the degree of hearing loss. The more severe the vestibular and cochlear EH, the more severe the hearing loss.

## Conclusions

3D-real IR MRI with intravenous gadolinium injection can provide a better assessment of EH in MD patients. The clinical features of MD patients with EH confirmed by enhanced MRI did not fully meet the existing diagnostic criteria for definite MD. Including the diagnosis of EH in the diagnostic criteria of MD can increase the diagnosis rate of MD. Besides, the degree and distribution of EH may be related to the degree of hearing loss.

## Funding

This work was supported by the 10.13039/501100003399Shanghai Municipal Science and Technology Commission Biomedicine Division Western Medicine Guidance Project (grant nº 19411965700).

## Ethics approval

The medical ethics committee of the EENT Hospital of Fudan University had approved this research (2020056).

## Authors’ contributions

W.C. and X.W. conceived and designed research; Y.G. collected data and conducted research; N.E.L. wrote the initial paper; Y.S. had primary responsibility for the final content. All authors read and approved the final manuscript.

## Conflicts of interest

The authors declare no conflicts of interest.
